# Cryptotanshinone protects skin cells from ultraviolet radiation-induced photoaging *via* its antioxidant effect and by reducing mitochondrial dysfunction and inhibiting apoptosis

**DOI:** 10.3389/fphar.2022.1036013

**Published:** 2022-10-25

**Authors:** Keke Guo, Run Liu, Rongrong Jing, Lusheng Wang, Xuenan Li, Kaini Zhang, Mengli Fu, Jiabin Ye, Zhenlin Hu, Wengang Zhao, Nuo Xu

**Affiliations:** ^1^ College of Life and Environmental Sciences, Wenzhou University, Wenzhou, China; ^2^ Zhiyuan College, Shanghai Jiao Tong University, Shanghai, China; ^3^ Department of Pharmacy, Zhoupu Hospital, Shanghai, China; ^4^ School of Medicine, Shanghai University, Shanghai, China; ^5^ Institute of Life Sciences, Wenzhou University, Wenzhou, China

**Keywords:** cryptotanshinone (CTS), ultraviolet (UV) radiation, photoaging, oxidative stress, mitochondrial dysfunction, apoptosis, mitochondrial synthesis

## Abstract

The integrity of skin tissue structure and function plays an important role in maintaining skin rejuvenation. Ultraviolet (UV) radiation is the main environmental factor that causes skin aging through photodamage of the skin tissue. Cryptotanshinone (CTS), an active ingredient mianly derived from the *Salvia* plants of Lamiaceae, has many pharmacological effects, such as anti-inflammatory, antioxidant, and anti-tumor effects. In this study, we showed that CTS could ameliorate the photodamage induced by UV radiation in epidermal keratinocytes (HaCaT) and dermal fibroblasts (HFF-1) when applied to the cells before exposure to the radiation, effectively delaying the aging of the cells. CTS exerted its antiaging effect by reducing the level of reactive oxygen species (ROS) in the cells, attenuating DNA damage, activating the nuclear factor E2-related factor 2 (Nrf2) signaling pathway, and reduced mitochondrial dysfunction as well as inhibiting apoptosis. Further, CTS could promote mitochondrial biosynthesis in skin cells by activating the AMP-activated protein kinase (AMPK)/sirtuin-1 (SIRT1)/peroxisome proliferator-activated receptor-γ co-activator-1α (PGC-1α) signaling pathway. These findings demonstrated the protective effects of CTS against UV radiation-induced skin photoaging and provided a theoretical and experimental basis for the application of CTS as an anti-photodamage and anti-aging agent for the skin.

## Introduction

The skin is the largest organ of the human body and it is composed mainly of two structural layers called the *epidermis*, the outer layer, and the dermis, the inner layer (Baroni et al., 2012). Skin aging is an inevitable physiological change that occurs over time, accompanied by phenotypic changes such as roughness, atrophy, and thinning of the skin, as well as the loss of skin elasticity and appearance of wrinkles. This aging process is closely related to the reduction of the number and function of keratinocytes and fibroblasts in the internal structure of the skin, as well as the reduction and atrophy of collagen and elastin in the dermis (Oldoni et al., 2016). Skin aging is caused by the interaction of internal and external factors in the body. In addition to the intrinsic aging caused by endogenous factors such as genome instability, telomere shortening, and accumulation of mitochondrial DNA (mtDNA) damage (Makrantonaki and Zouboulis, 2007), exposure of the skin to external environmental factors such as environmental toxins, pathogens and ultraviolet (UV) radiation can also lead to extrinsic aging of the skin and accelerate the overall aging process of the skin (Sanches Silveira and Myaki Pedroso, 2014). Among the external environmental factors that cause skin aging, UV radiation is the most important factor (Sanches Silveira and Myaki Pedroso, 2014). UV radiation is composed of the following three different types: Ultraviolet A (UVA) (320–400 nm), ultraviolet B (UVB) (280–320 nm), and ultraviolet C (UVC) (200–280 nm). About 95% UVA and about 5% UVB can penetrate the ozone layer and reach the ground (Fitsiou et al., 2021). UVA has long wavelengths and strong penetration, which enable it to be absorbed by the dermal cells (Oyewole et al., 2014). The energy of UVB is high but its wavelength is short, and therefore, it is mainly absorbed by the *epidermis* (Battie et al., 2014). There are 37 trillion cells in the human body, of which about two trillion exist in the skin (Bianconi et al., 2013). Generally, up to 41% of the body surface area is exposed to the Sun (Barger-Lux and Heaney, 2002). Theoretically, up to 820 billion skin cells are exposed to ultraviolet light (van Erp et al., 1996). Therefore, UV radiation will damage a large number of skin cells in the human body. UV radiation destroys key macromolecules in the skin, such as nucleic acids, lipids and proteins (Avadhani et al., 2017). In addition, UV radiation also causes skin erythema, inflammation, sunburn, immunosuppression, and skin cancer (Lephart, 2016; de Jager et al., 2017; Kawashima et al., 2018). Oxidative stress and mitochondrial dysfunction are important mechanisms leading to cellular aging (Passos et al., 2007; Birket et al., 2009). Under normal physiological conditions, reactive oxygen species (ROS) naturally generated by cellular respiration in the mitochondria play an important role in the regulation of cell signaling and intracellular homeostasis (Hudson et al., 2016). However, under environmental stress such as excess UV radiation, the ROS level in the cells can be significantly increased, resulting in oxidative stress (Anderson et al., 2014). On the one hand, UVA photon energy can be absorbed by intracellular photosensitizer chromophores such as porphyrin and bilirubin (Ravanat et al., 2001) or activate intracellular NADPH oxidase (NOX1) (Masaki et al., 1999), which also leads to the induction of ROS production. On the other hand, DNA in epidermal keratinocytes can directly absorb UVB and become damaged, which manifests in the forms of cyclobutane pyrimidine dimers (CPDs) and pyrimidine (6-4) pyrimidone photoproducts (6-4PPs) (Ravanat et al., 2001). Such DNA damage also leads to increased ROS production in the cells (Bosch et al., 2015). It has been reported that UV radiation-induced ROS can up-regulate the expression of intracellular senescence-associated β-galactosidase (SA-β-gal) (Park and Park, 2019; Zheng et al., 2021) and promote aging cells to continuously secrete senescence-associated secretory phenotype (SASP) factors such as pro-inflammatory factors [such as interleukin (IL)-6, IL-8] and matrix metalloproteinases (MMPs), induce skin inflammatory response and degradation of collagen, elastin, and fibronectin in the extracellular matrix, causing the skin to become thinner and wrinkle (Röck and Fischer, 2011). Cells can play an endogenous antioxidant function by up regulating the level of nuclear factor E2-related factor 2 (Nrf2) and its downstream antioxidant enzymes (Ben-Yehuda Greenwald et al., 2017). However, excessive ROS induced by high doses of UV may inactivate Nrf2 (Saw et al., 2011).

Mitochondria produce ATP through oxidative phosphorylation (OXPHOS) reactions in the mitochondrial electron transport chain (ETC) to provide energy for the cells (Sinha et al., 2013). When electrons are transferred along the ETC, 1%–3% of the electrons can escape and directly combine with O_2_ to produce O_2_^•−^, generating a small amount of ROS (Orrenius et al., 2007). The protons in the mitochondrial matrix are pumped out through the inner membrane into the intermembrane space, thereby establishing a proton gradient across the inner membrane, eventually giving rise to the mitochondrial membrane potential (MMP) (Sinha et al., 2013). Mitochondrial DNA is located in the mitochondrial matrix and is directly exposed to ROS produced by the ETC (Birch-Machin et al., 2013). This makes the mtDNA extremely vulnerable to damage by ROS. The damaged mtDNA is not easily repaired because of the lack of a repair system, and this can result in cumulative damage to the mtDNA (Makrantonaki and Zouboulis, 2007). Since mtDNA encodes the different protein components of the ETC, any damage sustained by the mtDNA could lead to mutations in these genes, resulting in dysfunctional mitochondria. In dysfunctional mitochondria, complexes II and III in the ETC produce single-electron leak sites, resulting in further increases in electron side leakage, which in turn leads to the reduction of MMP and ATP synthesis, as well as a surge in ROS level (Anderson et al., 2014). Besides, a decline in ATP level will lead to insufficient energy supply and reduced mitochondrial mass, eventually giving rise to a series of changes associated with aging of the body. It has been reported that AMP-activated protein kinase (AMPK) and sirtuin-1 (SIRT1) are important energy receptors in cells (Cantó and Auwerx, 2009), and activated AMPK can increase ATP production (Bergeron et al., 2001; Zong et al., 2002). SIRT1 normally cooperates with intracellular AMPK to promote mitochondrial biosynthesis and regulate mitochondrial homeostasis (Chang et al., 2015). Mitochondria are the regulatory hub of the apoptosis pathway, and UV radiation mainly induces skin cell apoptosis by activating the mitochondrial-linked apoptosis pathway (Chistiakov et al., 2014). Mitochondrial dysfunction caused by UV radiation will result in a decrease in ATP production, and this can destroy the regulatory mechanism of cellular calcium channels, with a subsequent impact on mitochondrial membrane potential and permeability, eventually leading to cell death or apoptosis (Liu et al., 2017). Therefore, reducing oxidative stress caused by UV radiation and improving mitochondrial function are the keys to delaying aging.

Cryptotanshinone (CTS) is a fat-soluble diterpenoid anthraquinone compound, which mainly exists in *Salvia* plants of Lamiaceae such as *Salvia miltiorrhiza* Bunge, *Salvia tebesana* Bunge and *Salvia przewalskii* Maxim (Wu et al., 2020). *Salvia miltiorrhiza* Bunge (Lamiaceae) is widely used in traditional Chinese medicine and current health care products (Zhou et al., 2005). The dried roots and rhizomes of *Salvia miltiorrhiza* Bunge (Lamiaceae) have a wide range of pharmacological effects, including anti-inflammatory, anti-oxidation, vasodilation, lowering of blood lipid, and improving energy metabolism among others (Cheng, 2006; Maione and Mascolo, 2016). CTS isolated from the dried roots and rhizomes of *S. miltiorrhiza* Bunge (Lamiaceae) has a strong antibacterial activity (Lee et al., 1999; Pang et al., 2016). CTS has potential applications in dermatology, where it plays an important role in the treatment of psoriasis (Tang et al., 2018), UV radiation-induced melanoma (Ye et al., 2016) and scar formation (Li et al., 2016). However, to the best of our knowledge, there has been no relevant report on the effect of CTS on UV radiation-induced skin aging. CTS has been shown to have strong antioxidant activity (Wang et al., 2007). CTS can also stabilize MMP, reduce ROS production and promote mitochondrial biosynthesis in cardiomyocytes (Zhang et al., 2016). In addition, CTS has been shown to prolong the life span of the budding yeast *Saccharomyces cerevisiae*, suggesting that CTS may also delay aging (Wu et al., 2014). These reports all suggest that CTS may delay skin aging by inhibiting UV radiation-induced oxidative damage and mitochondrial dysfunction in skin cells.

This study evaluated the beneficial effects of CTS on the delay of skin aging by using a human keratinocyte and fibroblast aging model established through UV radiation exposure. The potential mechanism underlying the impacts of CTS on skin cell oxidative damage, mitochondrial dysfunction, and apoptosis induced by UV radiation was also discussed.

## Materials and methods

### Materials

CTS was purchased from Shanghai Yuanye Bio-Technology Co., Ltd. (Shanghai, China).

### Cell lines and cell culture

The human keratinocyte cell line, HaCaT, was purchased from Sciencell (San Diego, California, United States), and the human skin fibroblast cell line, HFF-1, was obtained from the Cell Bank of the Chinese Academy of Sciences (Shanghai, China). HaCaT cells were cultured in Dulbecco’s modified Eagle’s medium (DMEM) (GIBCO, Life Technologies Corporation, NY, United States) supplemented with 10% fetal bovine serum (FBS) (GIBCO, Life Technologies Corporation, NY, United States) and 1% penicillin/streptomycin (Thermo Fisher Scientific, Waltham, MA, United States), whereas HFF-1 cells were cultured in DMEM containing 15% fetal bovine serum and 1% penicillin/streptomycin. Both cells were maintained at 37°C in a 5% CO_2_ incubator.

### Ultraviolet irradiation

HaCaT cells and HFF-1 cells were incubated without or with CTS (0.02, 0.05, and 0.1 μM) for different times according to the specific experiments. After that, the cells were washed with PBS and then kept in fresh PBS while being exposed to UV radiation in a UV irradiator (Vilber, Lourmat, Marne La Vallee, France). The control cells (non-irradiated cells) were covered with tinfoil while being exposed to UV radiation. For all UV irradiation experiments, HaCaT cells were irradiated by exposure to UVB (200 mJ/cm^2^) whereas HFF-1 cells were irradiated by exposure to UVA (15 J/cm^2^). After irradiation, the PBS was replaced with fresh DMEM containing no CTS or supplemented with the corresponding doses of CTS and incubated at 37°C in a 5% CO_2_ incubator. The incubation time was set according to the specific experiment.

### Senescence-associated β-galactosidase staining

HaCaT cells and HFF-1 cells were pretreated without and with CTS (0.02, 0.05, and 0.1 μM) for 12 h, washed with PBS, and then subjected to UV irradiation. After that, the PBS was removed and replaced with fresh medium containing no or the corresponding concentrations of CTS and incubated for 12 h in the case of HaCaT cells, and 24 h for HFF-1 cells. Subsequently, the cells were fixed in cold 4% paraformaldehyde for 5 min, washed with HBSS (Thermo Fisher Scientific, Waltham, MA, United States), stained with Hoechst 33342 (Dojindo Laboratories, Tokyo, Japan) for 15 min, and then incubated with SPiDER-βGal (Dojindo Laboratories, Tokyo, Japan) for 15 min. Finally, the fluorescence of SA-β-gal-positive cells was captured using a fluorescence inverted microscope (Zeiss, Germany) and the data were statistically analyzed by ImageJ software.

### Determination of reactive oxygen species in cells

HaCaT cells and HFF-1 cells pretreated without and with CTS (0.02, 0.05, and 0.1 μM) for 4 h were subjected to UV irradiation, washed twice with PBS, and then incubated with 10 μM DCFH-DA (Beyotime, Jiangsu, China) at 37°C in a 5% CO_2_ incubator for 20 min. After that, the cells were washed three times with a serum-free medium to fully remove the DCFH-DA that did not enter the cells. The cells were then digested with trypsin, harvested and washed with PBS. Finally, the cells were subjected to ROS assay as detected by a ROS assay kit (Beyotime, Jiangsu, China) with the aid of an ACEA NovoCyte flow cytometer (Agilent, Santa Clara, CA, United States) according to the manufacturer’s protocol. The data were analyzed using NovoExpress software.

### Immunofluorescence staining assay

HaCaT cells grown on coverslips (WHB Scientific, Shanghai, China) were pretreated without and with CTS (0.02, 0.05, and 0.1 μM) for 4 h, washed with PBS, and then subjected to UVB irradiation. After that, the PBS was replaced with fresh medium containing no CTS or the corresponding concentrations of CTS followed by 2 h of incubation. An immunofluorescence staining assay was performed as described in our previous study (Gao et al., 2021). HaCaT cells were incubated with anti-γ-H2AX (#9718, 1: 1000, Cell Signaling Technology, Beverly, MA, United States) and anti-CPD (CAC-NM-DND-001, 1: 1000, Cosmo Bio, Tokyo, Japan) at 4°C overnight. Subsequently, the cells were washed three times with TBST, and then incubated with Alexa Flour™ 488 donkey anti-rabbit secondary antibody (A21206, 1: 500, Thermo Fisher Scientific, Waltham, MA, United States) and Alexa Flour™ 568 donkey anti-mouse secondary antibody (A10037, 1: 500, Thermo Fisher Scientific, Waltham, MA, United States) at room temperature and in the dark for 2 h. Eventually, the coverslips were mounted on glass slides with an anti-fluorescence quencher containing DAPI (Thermo Fisher Scientific, Waltham, MA, United States). The DNA damage level in the cells was observed using a laser confocal microscope (Olympus, Tokyo, Japan) and the fluorescence data were statistically analyzed by ImageJ software.

### Mitochondrial mass analysis

The fluorescent probe Mito-Tracker Green (Beyotime, Jiangsu, China) was used to determine the mitochondrial mass of HaCaT cells and HFF-1 cells. HaCaT cells and HFF-1 cells were pretreated without and with CTS (0.02, 0.05, and 0.1 μM) for 4 h, washed with PBS, and then irradiated with UV light. Subsequently, the HaCaT cells were cultured with fresh complete medium containing no CTS or supplemented with the corresponding concentrations of CTS for 1 h before proceeding to the staining step while HFF-1 cells were subjected to staining without any incubation. The cells were then stained with 20 μM Hoechst dye (Dojindo Laboratories, Tokyo, Japan) at 37°C in the dark for 15 min. After that, the cells were washed three times with PBS and stained with 50 nM Mito-Tracker Green for 20 min. This was followed by three washes in PBS. Finally, the fluorescence of the mitochondria was measured with an inverted fluorescence microscope (Zeiss, Germany), and the fluorescence results were statistically analyzed using ImageJ software.

### Detection of mitochondrial membrane potential in cells by JC-1 staining

HaCaT and HFF-1 cells used to determine the presence of MMP following UV irradiation were treated as described for mitochondrial mass analysis. However, after the Hoechst staining step, the cells were washed with PBS, and then further stained with a mitochondrial membrane potential assay kit (JC-1) (Beyotime, Jiangsu, China) according to the manufacturer’s instruction. Finally, the presence of MMP was observed with a fluorescence inverted microscope (Zeiss, Germany) and the fluorescence results were statistically analyzed using ImageJ software, MMP = Red fluorescence intensity/Green fluorescence intensity.

### Measurement of intracellular ATP production

HaCaT cells and HFF-1 cells were pretreated without and with CTS (0.02, 0.05, and 0.1 μM) for 4 h, washed with PBS, and then irradiated with UV irradiation. The Enhanced ATP Assay Kit (Beyotime, Jiangsu, China) was used to detect the intracellular level of ATP. In brief, the cells were lysed with the cell lysis buffer provided in the kit, and the cell lysate was then centrifuged at 12,000 ×*g* for 5 min at 4°C. Subsequently, the clear supernatant was mixed with the ATP detection working solution in a 96-well plate. Finally, the relative luminescence unit (RLU) value was measured with a NanoQuant Plate (Tecan, Mannedorf, Switzerland) and the ATP concentration was calculated according to the manufacturer’s protocol.

### Calcein-AM/PI double staining assay

HaCaT cells and HFF-1 cells were pretreated without and with CTS (0.02, 0.05, and 0.1 μM) for 4 h, washed with PBS, and then subjected to UV irradiation. After that, the cells were incubated in a fresh medium containing no CTS or supplemented with the corresponding concentrations of CTS (0.02, 0.05, and 0.1 μM) for 6 h in the case of HaCaT cells, and 24 h for HFF-1 cells. Next, the cells were washed with PBS, digested with trypsin, and then centrifuged at 1000 ×*g* for 3 min. The cell pellet was resuspended in PBS and adjusted to a cell concentration of 10^5^–10^6^ cells/ml. After that, the cell suspension was incubated with 2.5 μM Calcein-AM/PI working solution (Dojindo Laboratories, Tokyo, Japan) for 15 min at 37°C, and then washed with PBS. Finally, the cells were resuspended in PBS and transferred onto a microscopic slide, and examined with a fluorescence inverted microscope (Zeiss, Germany). The fluorescence data were statistically analyzed using ImageJ software.

### RNA isolation and quantitative real-time PCR

The total RNA was isolated from HaCaT cells and HFF-1 cells using the GeneJET RNA Purification Kit (Thermo Fisher Scientific, Waltham, MA, United States) according to the manufacturer’s instructions. The concentration of total RNA was measured with a NanoQuant Plate (Tecan, Mannedorf, Switzerland). The obtained RNA was used as a template to synthesize the first cDNA strand, carried out with the PrimeScript RT Kit (Takara, Dalian, China). After obtaining cDNA, quantitative real-time PCR (qPCR) was performed on the LC96 system (Roche, Basel, Switzerland) using SYBR Green Master Mix (Applied Biosystems, Foster City, CA). The sequences of primers used in the qPCR are listed in Table 1. GAPDH mRNA was used as endogenous control for IL-6 mRNA, IL-8 mRNA, MMP-1 mRNA and MMP-3 mRNA, and COX1 mRNA was used as endogenous control for mtDNA mRNA. The relative expression of each target gene was analyzed using the 2^−ΔΔCt^ method. And the relative expression of mtDNA was used to determine the mtDNA copy number.

**TABLE 1:** Gene primer sequences for the quantitative real-time PCR.

Gene name	Primer sequences
matrix metalloproteinase-1 (MMP-1)	F:5′-TGGGCTGAAAGTGACTGGGAAAC-3′
	R:5′-ACATCTGGGCTGCTTCATCACC-3′
matrix metalloproteinase-1 (MMP-3)	F:5′-AGTTCCTTGGATTGGAGGTGACG-3′
	R:5′-TTCGGGATGCCAGGAAAGGTTC-3′
Interleukin-6 (IL-6)	F:5′-TCAATGAGGAGACTTGCCTGG-3′
	R:5′-GGGTCAGGGGTGGTTATTGC-3′
Interleukin-8 (IL-8)	F:5′- GAC​ATA​CTC​CAA​ACC​TTT​CCA​C-3′
	R:5′- CTT​CTC​CAC​AAC​CCT​CTG​C-3′
GAPDH	F:5′-ACAACTTTGGTATCGTGGAAGG-3′
	R:5′-GCCATCACGCCACAGTTTC-3′
mitochondrial DNA (mtDNA)	F:5′- CAC​CCA​AGA​ACA​GGG​TTT​GT-3′
	R:5′- TGG​CCA​TGG​GTA​TGT​TGT​TA-3′
COX1	F:5′- ACT​CCT​GCC​ATC​ATG​ACC​CTT​G-3′
	R:5′- TCG​GTT​GGT​CTC​TGC​TAG​TGT​G-3′

### Western blot

HaCaT cells and HFF-1 cells were pretreated without and with CTS (0.02, 0.05, and 0.1 μM) for 4 h, washed with PBS, and then subjected to UV irradiation. After that, the cells were incubated without or with the corresponding concentrations of CTS for 30 min after UV irradiation. After that, total proteins were extracted from the cells using Glo Lysis Buffer (Promega, Madison, WI, United States) added with Phosphatase Inhibitor Cocktail (bimake, Shanghai, China) and Protease Inhibitor Cocktail (bimake, Shanghai, China). Cytoplasmic and nuclear proteins were extracted from the cells using NE-PER™ Reagents (Thermo scientific, NY, United States). Total proteins, cytoplasmic proteins and nuclear proteins were then subjected to western blot performed as described in our previous study (Gao et al., 2021). The blot was incubated with primary antibody against caspase-3 (19677-1-AP, 1: 1,000, Proteintech, Wuhan, China), caspase-9 (66169-1-Ig, 1: 1,000, Proteintech, Wuhan, China), peroxisome proliferator-activated receptor-γ co-activator-1α (PGC-1α) (66369-1-Ig, 1: 1,000, Proteintech, Wuhan, China), Nrf2 (66504-1-Ig, 1: 1,000, Proteintech, Wuhan, China), heme oxygenase-1 (HO-1) (10701-1-AP, 1: 1,000, Proteintech, Wuhan, China), NAD(P)H: quinone oxidoreductase 1 (NQO1) (67240-1-Ig, 1: 1,000, Proteintech, Wuhan, China) or AMPK (#5832, 1: 1,000, Cell Signaling Technology, Beverly, MA, United States), phospho-AMPK (p-AMPK) (#2535, 1: 1,000, Cell Signaling Technology, Beverly, MA, United States), SIRT1 (#8469, 1: 1,000, Cell Signaling Technology, Beverly, MA, United States), Tubulin (#2148, 1: 1,000, Cell Signaling Technology, Beverly, MA, United States), LaminB (#12586, 1: 1,000, Cell Signaling Technology, Beverly, MA, United States) and probed with goat anti-rabbit antibody (A0208, 1: 2,000, Beyotime, Jiangsu, China) or goat anti-mouse antibody (A0216, 1: 2,000, Beyotime, Jiangsu, China). Detection of the blot was carried out with a chemiluminescence substrate (Thermo Scientific, NY, United States) and the image was captured with an Amersham Imager (GE Healthcare Biosciences, Pittsburgh, PA, United States) followed by the use of ImageJ software to analyze the result.

### Statistical analysis

All statistical analyses were performed using GraphPad Prism 6.0 (GraphPad, San Diego, CA, United States) software. One-way analysis of variance (ANOVA) was used to analyze the differences between groups followed by Dunnett’s test. All quantitative data were expressed as mean ± SD from three independent experiments, and statistical significance was considered at the *p* < 0.05 or *p* < 0.01 level.

## Results

### Protective effects of cryptotanshinone on HaCaT cells and HFF-1 cells senescence caused by ultraviolet radiation

**FIGURE 1:**
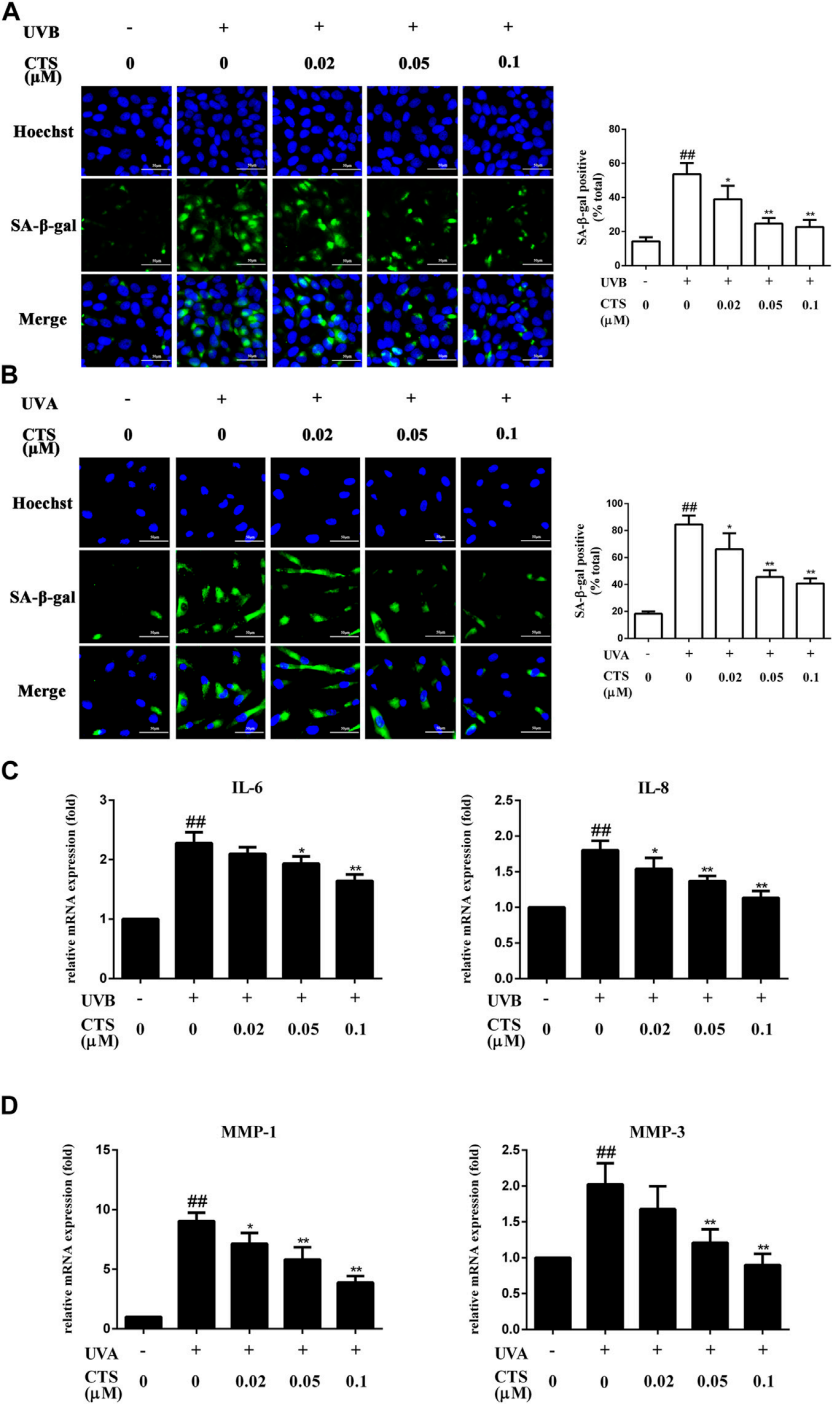
Effects of CTS on UV radiation-induced senescence in HaCaT cells and HFF-1 cells. HaCaT cells and HFF-1 cells were treated with CTS (0, 0.02, 0.05, 0.1 μM) for 12 h and then irradiated with UVB (200 mJ/cm^2^) and UVA (15 J/cm^2^), respectively. After that, the cells were cultured with fresh medium supplemented with the corresponding concentrations of CTS (0, 0.02, 0.05, and 0.1 μM) for another 12 h in the case of HaCaT cells before analysis, and 24 h for HFF-1 cells. **(A)** SA-β-gal-positive HaCaT cells as measured by fluorescence staining after 12-h of incubation following UVB irradiation. The plot besides the fluorescence image shows the number of senescent HaCaT cells. Scale bars: 50 μm. **(B)** SA-β-gal-positive HFF-1 cells as measured by fluorescence staining after 24-h of incubation following UVA irradiation. The plot besides the fluorescence image counts the number of senescent HFF-1 cells. Scale bars: 50 μm. **(C)** qPCR analysis of IL-6 and IL-8 mRNA levels in HaCaT cells after 12-h of incubation following UVB irradiation. **(D)** qPCR analysis of MMP-1 and MMP-3 mRNA levels in HFF-1 cells after 24-h of incubation following UVA irradiation. All data are mean ± SD from three independent experiments, “##” indicates significantly different from the control (no irradiation and CTS treatment) at the *p* < 0.01 level, whereas “*” and “**” indicate significantly different from the UV-irradiated cells without CTS pretreatment at the *p* < 0.05 and *p* < 0.01 levels, respectively.

SA-β-gal is overexpressed in senescent cells and is widely used as one of the markers of cell senescence (Campisi and d'Adda di Fagagna, 2007). UV-irradiated HaCaT cells exhibited significantly more SA-β-gal positive cells compared with the non-irradiated HaCaT cells. However, the number of SA-β-gal positive cells was significantly reduced when the cells were pretreated with CTS (0.02, 0.05, 0.1 μM) for 12 h before the UV irradiation (Figure 1A). Similarly, the number of SA-β-gal positive HFF-1 cells was significantly increased after UV irradiation compared with the non-irradiated HFF-1 cells. However, the number of SA-β-gal positive cells was significantly reduced when the HFF-1 cells were pretreated with CTS (0.02, 0.05, 0.1 μM) for 24 h before UV irradiation (Figure 1B). Senescent cells can affect the structure of skin tissue through the continuous secretion of SASP factors such as IL-6, IL-8 and MMPs, leading to a decline in tissue function (Röck and Fischer, 2011). Compared with the non-irradiated HaCaT cells, UV-irradiated HaCaT cells exhibited significantly increased IL-6 and IL-8 mRNA levels. Compared with UV-irradiated HaCaT cells, the mRNA level of IL-6 was significantly decreased in the cells pretreated with 0.05 and 0.1 μM of CTS before UVB irradiation, while the mRNA level of IL-8 was significantly decreased in the cells pretreated with 0.02, 0.05 and 0.1 μM of CTS before UV irradiation (Figure 1C). Consistently, the mRNA levels of matrix metalloproteinase (MMP)-1 and MMP-3 in UV-irradiated HFF-1 cells were significantly increased compared with the non-irradiated cells, while the increases in MMP-1 and MMP-3 mRNA levels induced by UV irradiation were attenuated in a dose-dependent manner in the cells pretreated with CTS (0.02, 0.05, 0.1 μM) before UVA irradiation (Figure 1D). These results showed that CTS exerted a protective effect against UV-induced senescence in HaCaT cells and HFF-1 cells.

### Cryptotanshinone attenuates ultraviolet radiation-inflicted oxidative damage in HaCaT and HFF-1 cells

**FIGURE 2:**
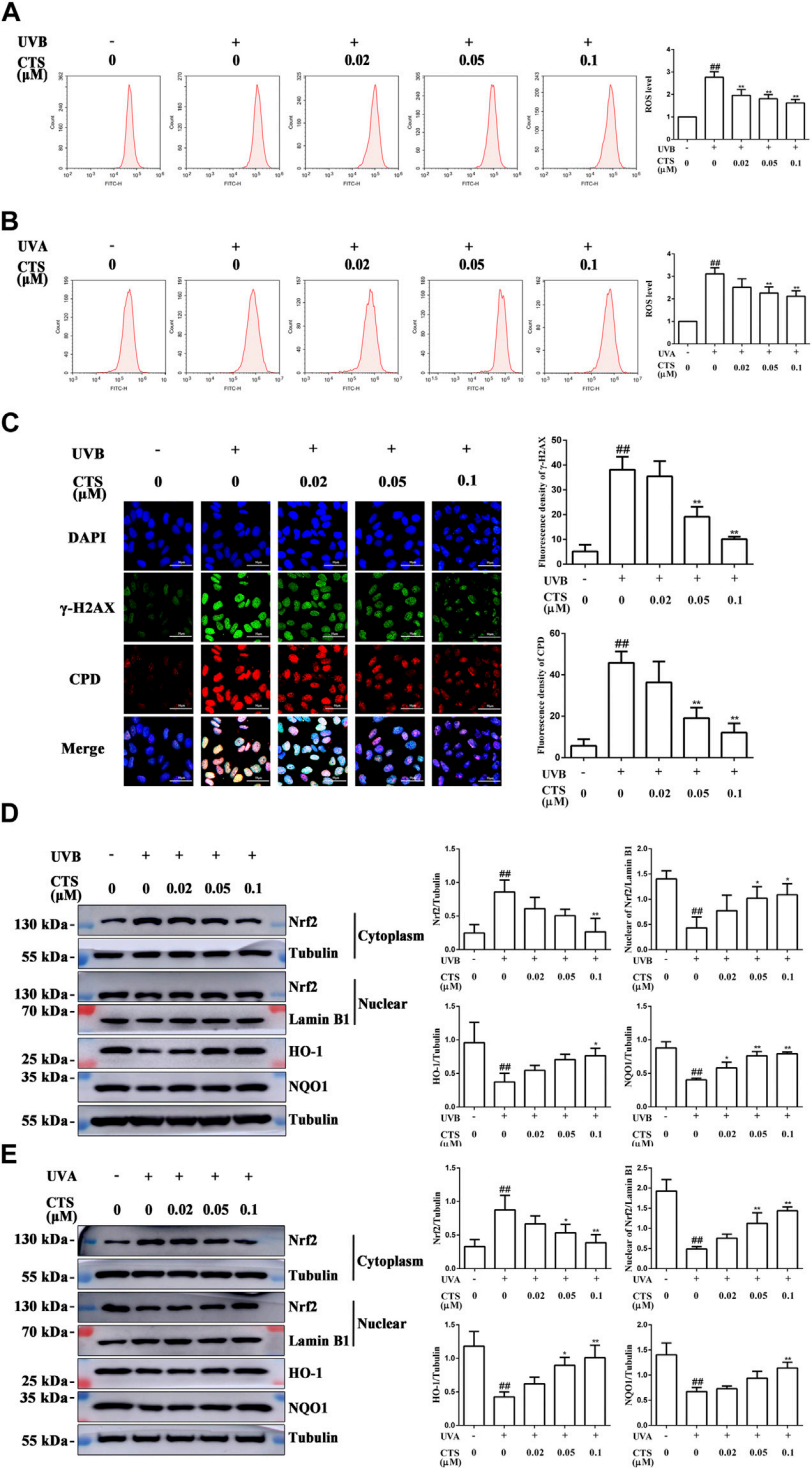
Effects of CTS on UV radiation-inflicted oxidative damage in HaCaT cells and HFF-1 cells. HaCaT cells and HFF-1 cells were treated with CTS (0, 0.02, 0.05, 0.1 μM) for 4 h prior to UVB (200 mJ/cm^2^) and UVA (15 J/cm^2^) irradiation, respectively. After UV irradiation, the cells were supplemented with the corresponding doses of CTS and incubated for different times before analysis. **(A)** Flow cytometry analysis of ROS production by HaCaT cells. The plot quantitatively compares the levels of ROS for the different CTS concentrations. **(B)** Flow cytometry analysis of ROS production by HFF-1 cells. The plot quantitatively compares the levels of ROS for the different CTS concentrations. **(C)** Immunofluorescence staining of CPD and γ-H2AX proteins in HaCaT cells after 2-h of incubation following UVB irradiation. Red fluorescence represents CPD, whereas green fluorescence represents γ-H2AX. All cells were counterstained with DAPI (blue). Scale bars: 50 μm. The plot besides the fluorescence image counts the number of CPD. Positive cells and γ-H2AX positive cells. **(D)** Western blot analysis of Nrf2, HO-1, NQO1 protein levels in the cytoplasm of HaCaT cells, and Nrf2 protein level in the nucleus after 0.5-h of incubation following UVB irradiation. The plots show the quantitative analysis of protein expression. **(E)** Western blot analysis of Nrf2, HO-1, NQO1 protein levels in the cytoplasm of HFF-1 cells, and Nrf2 protein level in the nucleus after 0.5-h of incubation following UVA irradiation. The plots show the quantitative analysis of protein expression. All data are mean ± SD from three independent experiments, “##” indicates significantly different from the control (no irradiation and CTS treatment) at the *p* < 0.01 level, whereas “*” and “**” indicate significantly different from the UV-irradiated cells without CTS pretreatment at the *p* < 0.05 and *p* < 0.01 levels, respectively.

The exposure of keratinocytes and fibroblasts to excessive UV radiation can cause oxidative stress and accelerate skin aging and the level of intracellular ROS is the most direct indicator of oxidative stress. HaCaT cells irradiated with UVB exhibited a significant increase in intracellular ROS level compared with the non-irradiated cells, but pretreatment of the cells with different concentrations of CTS before UVB irradiation led to a dose-dependent reduction in intracellular ROS level (Figure 2A). The same phenomenon was observed for HFF-1 cells in that pretreatment with CTS also reduced the intracellular ROS level (Figure 2B). These results indicated that CTS could reduce the surge of ROS production induced by UV radiation. The induction of intracellular ROS production is known to be triggered by irreversible DNA damage in epidermal keratinocytes after exposure to excessive UVB (Bosch et al., 2015), and CPD and γ-H2AX are two typical markers of photodamaged DNA (Huang et al., 2003; Zhao et al., 2014). The levels of CPD and γ-H2AX in HaCaT cells were increased when these cells were irradiated with UVB, but when the cells were pretreated with CTS (0.05, 0.1 μM) before the UVB irradiation, such an increase in either CPD or γ-H2AX was markedly reduced (Figure 2C). This suggested that CTS might prevent an increase in intracellular ROS production in HaCaT cells following UV irradiation by reducing the extent of DNA damage caused by the direct absorption of UVB.

It has been reported that Nrf2 is the main protein that a cell uses to resist oxidative stress (Kaspar et al., 2009). In the inactive state, Nrf2 combines with Nrf2-kelch-like ECH-associated protein 1 (Keap1) in the cytoplasm to promote its ubiquitination and proteasomal degradation. Once activated, Nrf2 separates from Keap1 and enters the nucleus where it then interacts with antioxidant response elements (AREs) and induce the expression of target genes, such as NQO1, HO-1 (Wang et al., 2016). HaCaT cells and HFF-1 cells both exhibited a significant decrease in the nuclear Nrf2 level following UV irradiation compared with the corresponding non-irradiated cells (Figures 2D,E). At the same time, the levels of HO-1 and NQO1 in these cells were also significantly decreased, indicating that UV radiation inhibited the activation of Nrf2 in skin cells and impaired its antioxidant capacity. Interestingly, both HaCaT and HFF-1 cells pretreated with CTS before UV irradiation showed increased levels of HO-1, NQO1, and nuclear Nrf2 compared with the UV-irradiated cells (Figures 2D,E), suggesting that CTS could promote the translocation of Nrf2 from the cytoplasm to the nucleus and its subsequent activation. These results suggested that following the UV irradiation of HaCaT and HFF-1 cells, CTS could alleviate the extent of oxidative damage by reducing the increase in ROS production and activating the Nrf2 signaling pathway in both types of cells.

### Cryptotanshinone reduces mitochondrial dysfunction in HaCaT cells and HFF-1 cells caused by ultraviolet radiation

**FIGURE 3:**
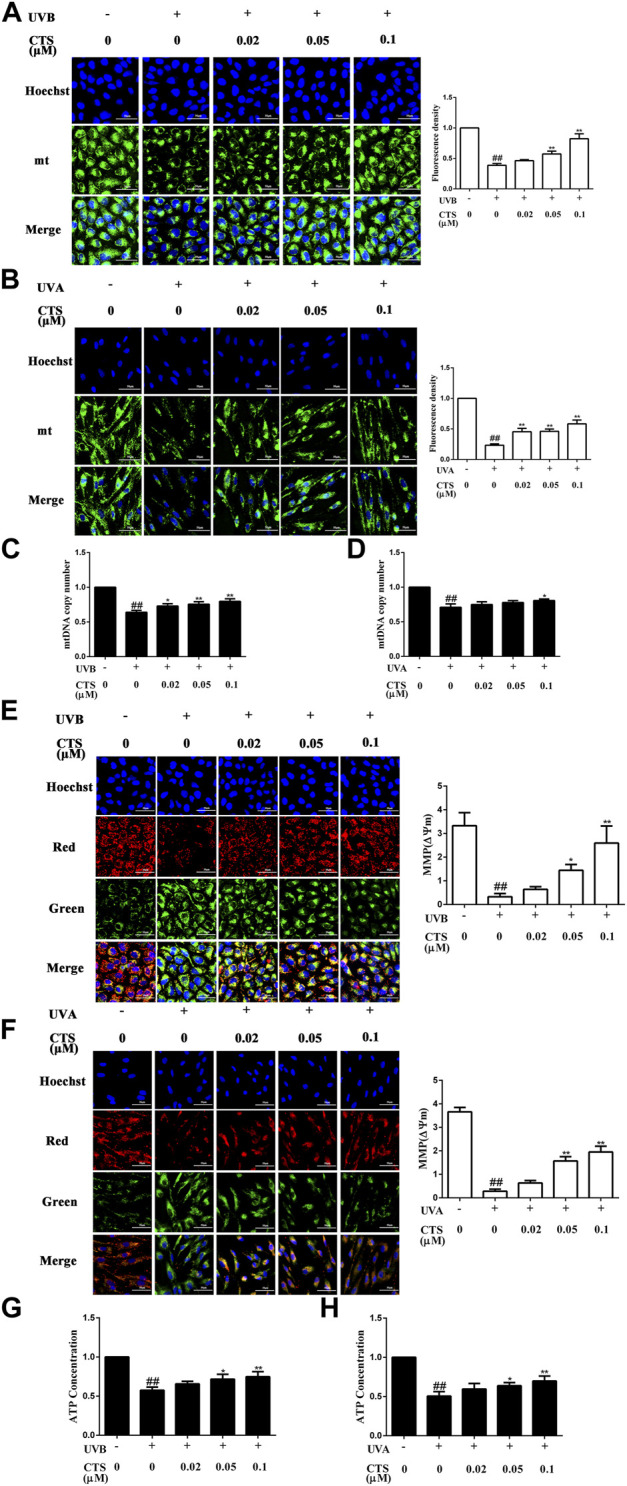
Effects of CTS on mitochondrial function in UV-irradiated HaCaT cells and HFF-1 cells. HaCaT cells and HFF-1 cells were treated with CTS (0, 0.02, 0.05, 0.1 μM) for 4 h and then irradiated with UVB (200 mJ/cm^2^) and UVA (15 J/cm^2^), respectively. After UV irradiation, the cells were supplemented with the corresponding concentrations of CTS and incubated for the different times before analysis. **(A)** Fluorescence staining of Mito-Tracker Green probed HaCaT cells after 1-h of incubation following UVB irradiation. Scale bars: 50 μm. The plot shows the quantitation of mitochondrial mass as determined by intensity analysis of the fluorescence in HaCaT cells. **(B)** Fluorescence staining of Mito-Tracker Green probed HFF-1 cells after UVA irradiation. Scale bars: 50 μm. The plot shows the quantitation of mitochondrial mass as determined by the intensity analysis of the fluorescence in HFF-1 cells. **(C)** Copy number of mtDNA in HaCaT cells as determined by qPCR. **(D)** Copy number of mtDNA in HHF-1 cells as determined by qPCR. **(E)** JC-1 staining of HaCaT cells after 1-h of incubation following UVB irradiation. Scale bars: 50 μm. The ratio of red to green fluorescence intensity represents MMP. The plot shows the change in MMP level in HaCaT cells. **(F)** JC-1 staining of HFF-1 cells after UVA irradiation. Scale bars: 50 μm. The ratio of red to green fluorescence intensity represents MMP. The plot shows the change in MMP level in HFF-1 cells. **(G)** ATP level in HaCaT cells following UVB irradiation as detected by Multi-Mode Microplate Reader. **(H)** ATP level in HFF-1 cells following UVA irradiation as detected by Multi-Mode Microplate Reader. All data are mean ± SD from three independent experiments, “##” indicates significantly different from the control (no irradiation and CTS treatment) at the *p* < 0.01 level, whereas “*” and “**” indicate significantly different from the UV-irradiated cells without CTS pretreatment at the *p* < 0.05 and *p* < 0.01 levels, respectively.

UV irradiation can result in mitochondrial dysfunction, which in turn can cause premature skin aging. It has been reported that partial recovery of mitochondrial function can rejuvenate aging skin (Singh et al., 2018). In this study, the effect of CTS on mitochondrial function was demonstrated by measuring the mitochondrial mass, mtDNA copy number, MMP and intracellular ATP level. The mitochondrial mass was significantly reduced in UV irradiated HaCaT and HFF-1 cells while treating these cells with CTS before irradiation could up-regulate the mitochondrial mass in a dose-dependent manner, as revealed by mitochondrial fluorescence staining (Figures 3A,B). Consistent with the results of fluorescence staining, the qPCR analysis revealed a decreasing copy number of mtDNA in the UV-irradiated cells compared with the non-irradiated cells (Figures 3C,D). Pretreatment of HaCaT cells with CTS (0.02, 0.05, 0.1 μM) was able to increase the mtDNA copy number in the cells in a dose-dependent manner following UV irradiation (Figure 3C), while only pretreatment of HFF-1 cells with 0.1 μM of CTS was able to increase the mtDNA copy number in HFF-1cells following UV irradiation (Figure 3D). The results indicated that CTS could attenuate the reduction of mtDNA copy number and mitochondrial mass in HaCaT cells and HFF-1 cells induced by UV radiation.

The reduction of MMP is a very important indicator of mitochondrial damage. Cells subjected to UV irradiation showed increased green fluorescence intensity and decreased red fluorescence intensity compared with non-irradiated cells (Figures 3E,F). The decreased ratio of red to green fluorescence intensity indicated a reduction in MMP in the UV irradiated cells compared with the non-irradiated cells. However, the decrease in MMP level induced by UV radiation in HaCaT and HFF-1 cells was considerably reduced when the cells were pretreated with CTS, and the higher the concentration of CTS, the stronger it could counteract the effect of UV radiation. Likewise, compared with the non-irradiated cells, the UV-irradiated cells exhibited significantly reduced ATP production, but pretreatment of the irradiated cells with CTS (0.05 and 0.1 μM) was able to significantly raise the production of ATP (Figures 3G,H). Taken together, the data suggested that CTS could reduce the mitochondrial dysfunction in HaCaT and HFF-1 cells caused by UV radiation.

### Cryptotanshinone effectively inhibits ultraviolet radiation-induced apoptosis in HaCaT cells and HFF-1cells

**FIGURE 4:**
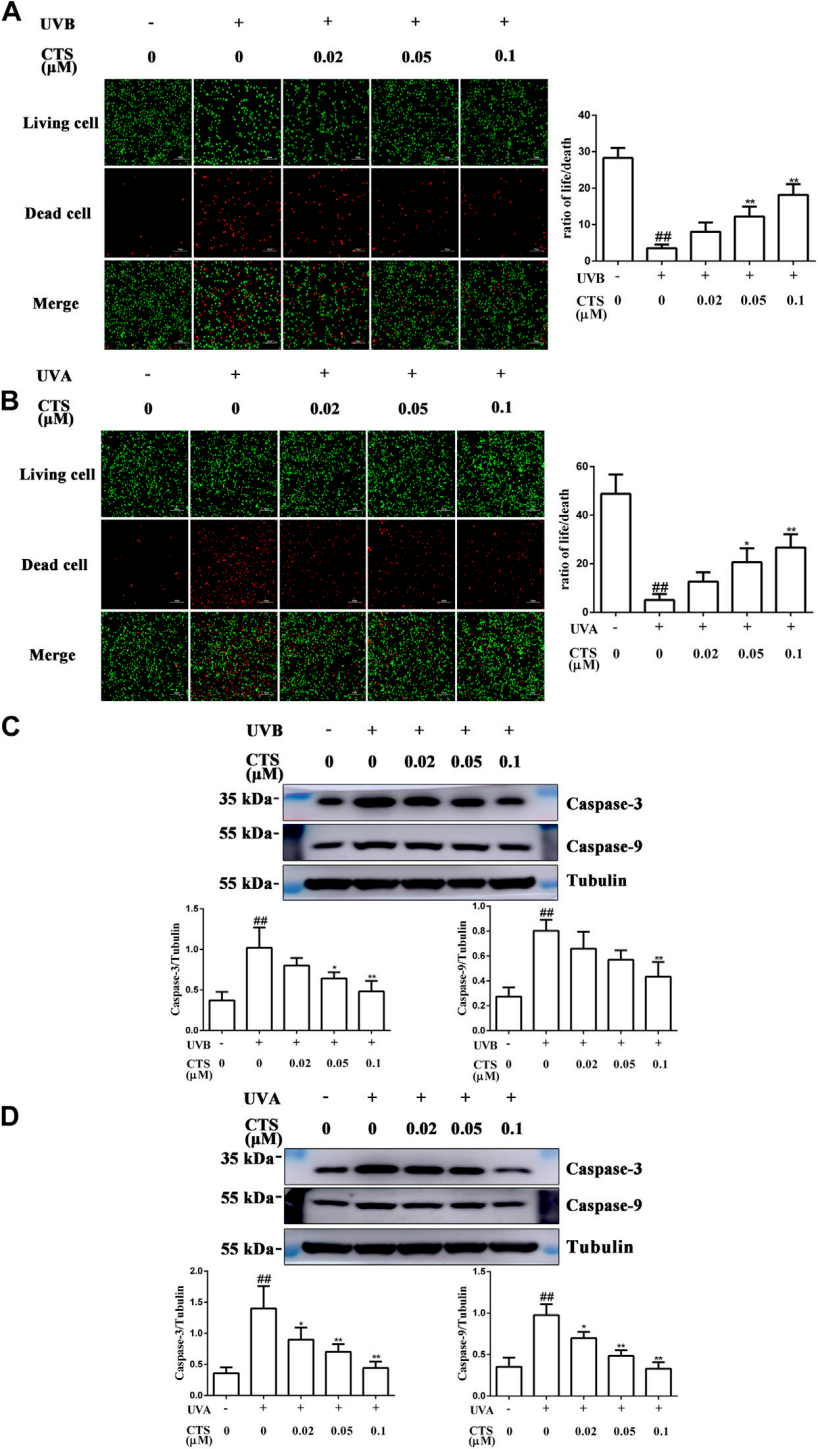
Effects of CTS on UV radiation-induced apoptosis in HaCaT cells and HFF-1 cells. HaCaT cells and HFF-1 cells were treated without and with CTS (0.02, 0.05, 0.1 μM) for 4 h prior to UVB (200 mJ/cm^2^) and UVA (15 J/cm^2^) irradiation, respectively. After UV irradiation, the cells were supplemented with the corresponding concentrations of CTS and incubated for different times before analysis. Calcein-AM/PI double staining of HaCaT cells after incubating for 6 h following UVB irradiation **(A)** and HFF-1 cells after incubating for 24 h following UVA irradiation **(B)**. Red fluorescence represents the dead cells, whereas green fluorescence represents the live cells. Scale bars: 100 μm. The plots show the ratio of living to dead cells. Caspase-3 and caspase-9 levels in HaCaT cells **(C)** and HFF-1 cells **(D)** after incubating 0.5 h following UV irradiation as assayed by western blot. The plots show the quantitative analysis of protein expression. All data are mean ± SD from three independent experiments, “##” indicates significantly different from the control (no irradiation and CTS treatment) at the *p* < 0.01 level, whereas “*” and “**” indicate significantly different from the UV-irradiated cells without CTS pretreatment at the *p* < 0.05 and *p* < 0.01 levels, respectively.

When the skin is exposed to excessive UV irradiation, it can result in the apoptosis of skin cells. For both HaCaT and HFF-1 cells, the number of dead cells increased significantly after UV irradiation compared with the corresponding non-irradiated cells. The ratio of living cells to dead cells also decreased for both cell types. However, the number of UV radiation-induced dead cells decreased when the cells were pretreated with CTS, and in a dose-dependent manner (Figures 4A,B). Furthermore, compared with the non-irradiated cells, the levels of caspase-3 and caspase-9 were increased in the UV-irradiated cells. However, the levels of caspase-3 and caspase-9 in the cells pretreated with CTS were reduced compared with the non-CTS-treated cells following UV irradiation, and the reduction was dependent on the concentration of CTS (Figures 4C,D). The results demonstrated that CTS could effectively inhibit UV radiation-induced apoptosis in HaCaT cells and HFF-1 cells.

### Cryptotanshinone promotes mitochondrial biosynthesis through activating the AMPK/SIRT1/PGC-1α signaling pathway

**FIGURE 5:**
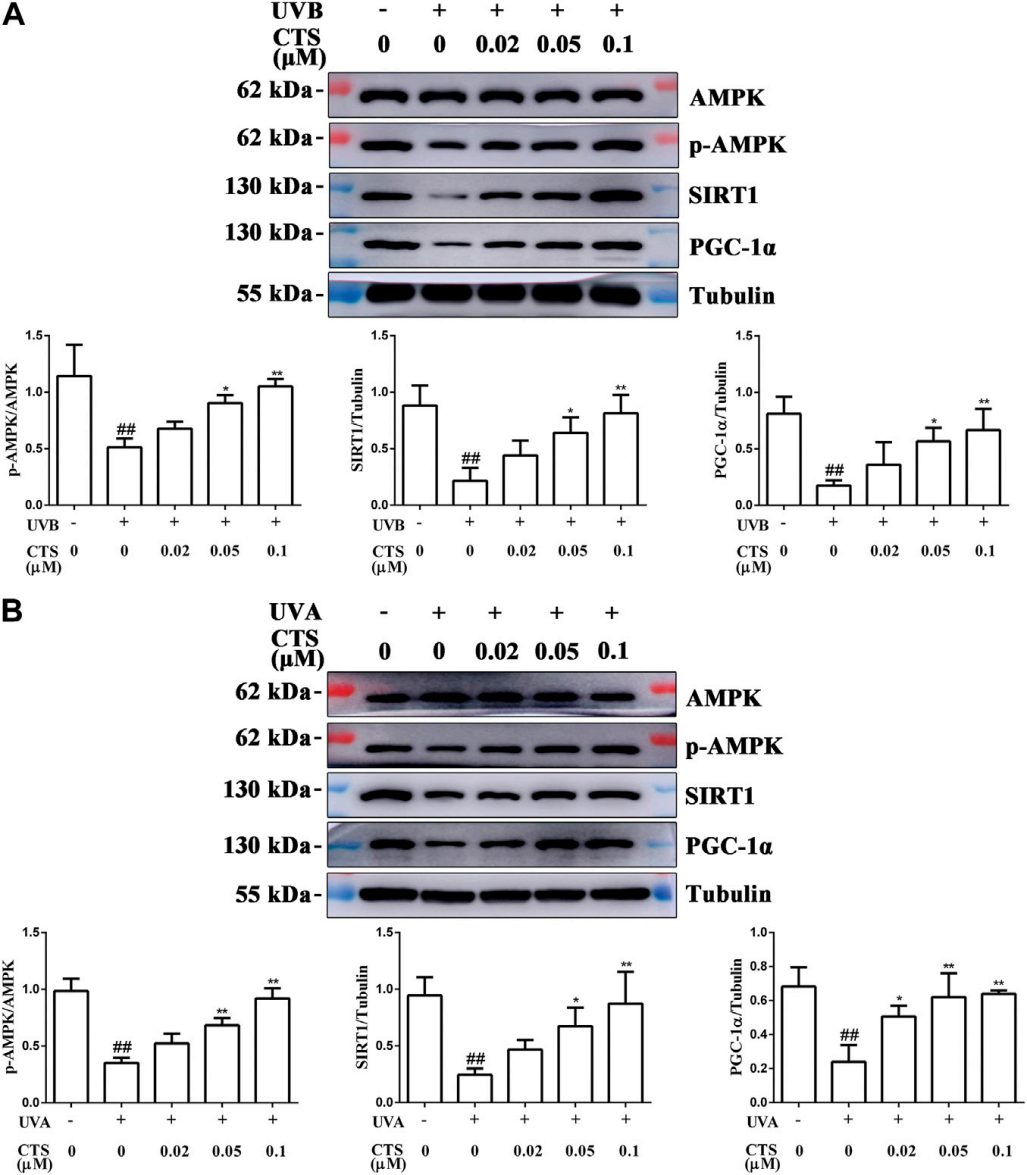
CTS promotes mitochondrial biosynthesis *via* the AMPK/SIRT1/PGC-1α signaling pathway. HaCaT cells and HFF-1 cells were treated with CTS (0, 0.02, 0.05, 0.1 μM) for 4 h and irradiated with UVB (200 mJ/cm^2^) or UVA (15 J/cm^2^), respectively. Then, the cells were supplemented with the corresponding doses of CTS and incubated for another 0.5 h before analysis. **(A)** Protein levels of p-AMPK, SIRT1 and PGC-1α in HaCaT cells as measured by western blot. The plots beside the blots show the relative expression levels of p-AMPK, SIRT1 and PGC-1α as determined by grayscale analysis of the bands in the blots. **(B)** Protein levels of p-AMPK, SIRT1 and PGC-1α in HFF-1 cells as measured by western blot. The plots beside the blots show the relative expression levels of p-AMPK, SIRT1 and PGC-1α as determined by grayscale analysis of the bands in the blots. All data are mean ± SD from three independent experiments, “##” indicates significantly different from the control (no irradiation and CTS treatment) at the *p* < 0.01 level, whereas “*” and “**” indicate significantly different from the UV-irradiated cells without CTS pretreatment at the *p* < 0.05 and *p* < 0.01 levels, respectively.

To further explore the mechanism by which CTS reduced mitochondrial function, HaCaT cells and HFF-1 cells were incubated without or with CTS (0.02, 0.05 and 0.1 μM) for 4 h before UV irradiation, and the intracellular levels of AMPK, p-AMPK, SIRT1 and PGC-1α were detected by western blot. UVB-irradiated HaCaT cells exhibited reduced levels of p-AMPK, SIRT1 and PGC-1α compared with the non-irradiated cells. However, when the cells were treated with CTS (0.05, 0.1 μM) before UV irradiation, the levels of p-AMPK, SIRT1 and PGC-1α in the cells increased significantly (Figure 5A). Moreover, decreases in the levels of p-AMPK, SIRT1 and PGC-1α induced by UVA irradiation in HFF-1 cells were suppressed when the cells were pretreated with CTS (0.02, 0.05, 0.1 μM) before UVA irradiation (Figure 5B). These findings suggested that CTS could promote mitochondrial biosynthesis *via* the AMPK/SIRT1/PGC-1α signal pathway, which would then reduce the UV radiation-induced mitochondrial dysfunction.

## 4 Discussion

In the process of aging, skin aging is the most obvious and intuitive feature because it has a significant negative impact on people’s appearance. Skin is the first barrier between the body and the external environment, and therefore, it is vulnerable to bombardment by excessive UV radiation. Long-term exposure of skin to UV radiation can disrupt tissue homeostasis, accelerate the onset of aging-related phenotypes, and increase the risk of skin cancer (Fitsiou et al., 2021). In recent years, the Earth has been exposed to increasing amounts of UV radiation because of the destruction of the atmosphere. Therefore, the protection from skin injury caused by UV radiation is becoming more important. The skin is composed mainly of two layers: the *epidermis* and the dermis (Baroni et al., 2012). The wavelength of UVB is short and UVB is mainly absorbed by the *epidermis*, resulting in skin sunburn, epidermal hyperplasia, and inflammatory reactions (Amaro-Ortiz et al., 2014; Battie et al., 2014), whereas UVA has long wavelengths and can penetrate deep into the dermis, making it a more significant factor in the induction of skin photoaging (Zarei and Abbaszadeh, 2019). Keratinocytes account for 90%–95% of epidermal cells and are the main target cells of UVB radiation (Tang et al., 2021). Fibroblasts are the main cells of dermis (Wong et al., 2016) and the main target cells of UVA radiation. Changes in the skin after exposure to UV radiation are related to the changes that occur at the cellular level. Therefore, in this study, UVB-irradiated HaCaT cells and UVA-irradiated HFF-1 cells were used as *in vitro* models to explore the effects of CTS on UV radiation-induced epidermal keratinocytes and dermal fibroblasts, respectively. CTS is a fat-soluble diterpenoid anthraquinone compound mainly existing in *Salvia* plants of Lamiaceae (Wu et al., 2020), and it plays an important role in UV-induced melanoma, reducing scar formation and treating psoriasis (Li et al., 2016; Ye et al., 2016; Tang et al., 2018). However, there has been no relevant report on the delay of skin aging by CTS. Our findings demonstrated that CTS can ameliorate UV radiation-induced skin aging by inhibiting oxidative stress, mitochondrial dysfunction and apoptosis.

In photoaging skin, aging cells continue to accumulate and secrete pro-inflammatory cytokines (Wang and Dreesen, 2018), MMPs and other SASP factors. This promotes the thinning of the *epidermis* and the degradation of collagen and elastic fibers in the extracellular matrix of the dermal tissue, thus accelerating the aging process of skin tissue (Fisher et al., 2002). It has been reported that CTS can decrease the activity of SA-β-gal in IL-10-induced activated hepatic stellate cells (HSCs), and such action of CTS can inhibit the senescence of HSCs (Huang et al., 2020). Our data clearly showed that CTS could inhibit UV radiation-induced skin photoaging. SA-β-gal-positive cells and SASP factors (IL-6, IL-8, MMP-1, MMP-3) in HaCaT cells and HFF-1cells were increased significantly when these cells were exposed to UV radiation, but these increases were significantly suppressed when the cells were treated with CTS before exposure to the UV radiation (Figure 1).

UV radiation-mediated oxidative stress is the key trigger of skin photoaging. The antioxidant mechanism of the active components of *S. miltiorrhiza* Bunge (Lamiaceae) comprises two main aspects: Direct scavenging of ROS and increased expression of antioxidant enzymes *via* the activation of the Nrf2 pathway (Zhao et al., 2008; Li et al., 2018). Our data demonstrated that 0.02–0.1 μM of CTS could effectively reduce the level of UV radiation-induced ROS production in HaCaT cells and HFF-1 cells (Figures 2A,B). UVB can be directly absorbed by DNA in epidermal keratinocytes, resulting in DNA damage, which indirectly leads to rising ROS levels. When the energy of a UVB photon is directly absorbed by DNA, cycloaddition occurs between the C5 and C6 of two adjacent pyrimidine bases in the DNA, converting them into CPD. The C6 and C4 of two adjacent pyrimidine bases form a covalent bond to generate 6–4 PP (Cadet et al., 2015). 6–4 PP mainly destroys the double helix structure of DNA, which induces the production of γ-H2AX (Hanasoge and Ljungman, 2007; Chen et al., 2014). An increase in CPD level will also induce more γ-H2AX formation. Therefore, the degree of DNA damage can be known by detecting the increases in CPD and γ-H2AX (Zhao et al., 2014). CTS showed a good anti-DNA damage effect as evidenced by the decreased CPD and γ-H2AX levels in HaCaT cells pretreated with CTS before UVB irradiation compared with the irradiated cells without CTS pretreatment (Figure 2C). Such a feature indicated that CTS could further reduce the ROS level in the cells by alleviating UV radiation-induced DNA damage to ameliorate the associated skin aging process.

Previous studies have shown that Nrf2 plays a beneficial role in protecting the skin from UVB induced inflammation, oxidative damage, cell dysfunction and skin sunburn (Saw et al., 2011; Ikehata and Yamamoto, 2018). The deficiency of Nrf2 will aggravate the UVB induced skin damage, such as inflammation, DNA damage and extracellular matrix damage (Saw et al., 2014; Knatko et al., 2015), while the activation of Nrf2 can prevent skin cancer caused by ultraviolet light (Li et al., 2017; Gęgotek et al., 2018; Rojo de la Vega et al., 2018). Cells regulate the occurrence of various oxidative stress injuries through the Nrf2 signaling pathway (Ben-Yehuda Greenwald et al., 2017). CTS has been found to exert various protective effects by activating the intracellular Nrf2 signaling pathway, such as alleviating ethanol-induced liver injury (Nagappan et al., 2019), regulating the endotoxin-induced inflammatory response of microglia (Zhou et al., 2019), and inhibiting the MG132-induced apoptosis (Lee et al., 2020). Since CTS could effectively inhibit UV radiation-induced ROS production in HaCaT and HFF-1 cells, we speculated that it may also inhibit oxidative damage to the skin cells induced by UV radiation through the Nrf2 signaling pathway. To verify this hypothesis, the effect of CTS on the levels of Nrf2 and its downstream antioxidant enzymes HO-1 and NQO1 was investigated by western blot. Surprisingly, CTS could promote the translocation of Nrf2 from the cytoplasm to the nucleus, enabling Nrf2 to transcriptionally activate its downstream antioxidant genes such as HO-1 and NQO1. Thus, CTS might achieve its antioxidant effect by activating the Nrf2 signaling pathway, thereby ameliorating photoaging caused by UV irradiation-induced oxidative stress.

Mitochondrial dysfunction can further induce cellular aging (Wiley et al., 2016). As mitochondria are the main site of ROS generation, the mtDNA is easily attacked by excessive ROS caused by UV irradiation (Birch-Machin et al., 2013), resulting in abnormal function for the ETC, with the consequence of promoting premature skin aging (Krutmann and Schroeder, 2009). Previous studies have shown that the copy number of mtDNA and the mitochondria mass will decrease with age (Zhang et al., 2017), and the damaged mitochondria will also be removed through the mitophagy mechanism (Li et al., 2020; Zhuang et al., 2020). Mitochondria play a central role in cellular energy metabolism and a range of other cellular activities, such as calcium signaling, iron homeostasis, hormone synthesis, and programmed cell death (Schon et al., 2012; Stewart and Chinnery, 2015; Picard et al., 2016). However, the loss of mitochondria will seriously affect the normal progress of cell activities. The mitochondrial DNA polymerase gamma gene (*POLG1*) is a nuclear gene that encodes the catalytic subunit of the mtDNA polymerase gamma, an enzyme that is essential for mtDNA replication (Milone and Massie, 2010). One study has described the construction of an inducible mtDNA-depleted mouse expressing a dominant-negative (DN) mutation in the polymerase domain of POLG1 (Singh et al., 2018). When the POLG1-DN transgene was turned on by doxycycline (dox), these transgenic mice showed reduced mtDNA content and obvious skin aging symptoms. However, when dox was withdrawn, the content of mtDNA in the mtDNA-depleter mice was restored to the normal level and the aging-related manifestations such as skin wrinkles and hair loss were reversed. This suggests that mtDNA is a reversible regulator of skin aging and the skin aging symptoms can, therefore, be reversed by restoring the normal level of mtDNA in the cells. Our data showed that CTS could significantly inhibit the reduction of mtDNA copy number and mitochondrial mass induced by UV radiation (Figures 3A–D). Since a decrease in mtDNA copy number will lead to a decrease in MMPs (Singh et al., 2018), our observation suggested that CTS may exert its anti-aging role by increasing MMP levels, possibly by increasing the copy number of mtDNA. Aging is related to the ability of mitochondria to produce energy (Prinzinger, 2005). Mitochondria are very important organelles in the cytoplasm of eukaryotic cells, which undertake a variety of important physiological functions. They mainly generate ATP through OXPHOS to provide energy for the cells, and hence the entire organism (Krutmann and Schroeder, 2009). The site of ATP synthesis is located in the ETC of the inner mitochondrial membrane (Hamanaka and Chandel, 2009). Mutation and reduction of mtDNA can lead to a defect in the ETC, which can easily result in decreased MMP and ATP levels. CTS has been shown to significantly promote the production of ATP and increase MMP levels by increasing the activities of all the complexes in the mitochondrial electron transport chain except for complex II, thereby playing a protective role in cardiomyocytes (Zhang et al., 2016). As CTS pretreatment also led to increased MMP and ATP levels in HaCaT cells and HFF-1 cells (Figures 3E–H), CTS may reduce mitochondrial damage by reducing the production of ROS and or by restoring the ETC function by preventing a drop in the copy number of mtDNA induced by UV radiation in this case, with the consequence of promoting ATP synthesis and restoring MMP level.

Moreover, excessive damage to the mitochondria induced by UV radiation can lead to an increase in mitochondrial outer membrane permeability (MOMP), which will allow cytochrome c (Cyt c) to leak from the mitochondria into the cytoplasm (Sinha et al., 2013). In the cytoplasm, Cyt c polymerizes with apoptotic protease activating factor-1 (Apaf-1), and this complex then activates caspase-3 and caspase-9, eventually causing apoptosis (Chipuk et al., 2010; Vo and Letai, 2010; Edlich et al., 2011). Thus, one way to prevent such apoptosis is to block the activation of these caspases, which are acting as executioners of apoptosis. By preventing an increase in the levels of active caspase-3 and caspase-9, CTS was able to effectively reduce the extent of apoptosis in HaCaT and HFF-1 cells induced by UV radiation. Interfering with the activation of executioner caspases, therefore, might constitute an important mechanism by which CTS could protect skin cells from photoaging.

In the case of oxidative stress, cells need to remove damaged mitochondria in time to maintain the normal structure and homeostasis (Sedlackova and Korolchuk, 2019). However, once the mass of mitochondria is reduced, it cannot meet the energy demand of cell metabolism, and this can also lead to premature aging of the skin. When this happens, it is necessary to activate the mitochondrial biosynthesis pathway so that new mitochondria can replace the lost ones. Mitochondrial biosynthesis is a key component in the mitochondrial mass control mechanism, and AMPK/SIRT1/PGC-1α is a key signaling pathway controlling mitochondrial biosynthesis. SIRT1 is an NAD^+^-dependent deacetylase. When NAD^+^ is increased in cells, the Thr 522 residue of SIRT1 is activated by phosphorylation (Cantó and Auwerx, 2009; Guo et al., 2010; Guo et al., 2012). SIRT1 has been shown to have anti-aging effects in various cell models, and its anti-aging effects are associated with the alleviation of intracellular oxidative stress (Wang et al., 2011; Cueno et al., 2014). PGC-1α can be deacetylated by SIRT1 to regulate its transcriptional activity (Cantó et al., 2010), and deacetylated PGC-1α enhances the replication of mtDNA by activating the mitochondrial biosynthesis pathway (Uittenbogaard and Chiaramello, 2014). A study has reported that CTS can effectively improve the expression level of PGC-1α and promote mitochondrial biosynthesis in oxidatively damaged cardiomyocytes (Zhang et al., 2016). SIRT1 normally cooperates with intracellular AMPK in the activation of PGC-1α (Chang et al., 2015). AMPK induces the phosphorylation of PGC-1α, while SIRT1 promotes the deacetylation of PGC-1α. In the UV-irradiated HaCaT cells and HFF-1 cells, the levels of p-AMPK, SIRT1 and PGC-1α were decreased significantly (Figure 5). Excessive UV radiation may inhibit the expression of p-AMPK, SIRT1, and PGC-1α by damaging nuclear DNA, thereby inhibiting mitochondrial biosynthesis. However, pretreatment of the cells with CTS significantly increased the p-AMPK, SIRT1 and PGC-1α levels in the UV-irradiated skin cells and activated the AMPK/SIRT1/PGC-1α pathway. Therefore, CTS might protect the mitochondrial function in skin cells against the damaging effect of UV radiation in two ways. The first way is by reducing the extent of mitochondrial damage *via* decreasing intracellular ROS levels. The second way is by activating the AMPK/SIRT1/PGC-1α pathway, a key signal pathway that initiates mitochondrial biosynthesis and generates new mitochondria. Although we have demonstrated, for the first time, the anti-aging effect of CTS on skin cells, and CTS shown that this effect involved the attenuation of oxidative damage, amelioration of mitochondrial dysfunction, and inhibition of cell apoptosis, whether CTS can play a role in delaying skin aging at an animal level remains a topic for further study.

## 5 Conclusion

In summary, CTS was found to activate the Nrf2-mediated antioxidant signaling pathway as demonstrated in UV-irradiated skin cells, with the result of inhibiting ROS generation and attenuating DNA damage. Moreover, CTS also reduced mitochondrial dysfunction and promoted mitochondrial biosynthesis by activating the AMPK/SIRT1/PGC-1α pathway. These effects of CTS would eventually lead to reduced cell death and the relief of photoaging. Besides, these effects of CTS could also establish a solid foundation for the application of CTS in anti-photoaging skin. CTS could indeed be considered a valuable component in the development of UV-protective agents.

## Data Availability

The original contributions presented in the study are included in the article/Supplementary Material, further inquiries can be directed to the corresponding authors.

## References

[B1] Amaro-Ortiz A. Yan B. D'Orazio J. A. (2014). Ultraviolet radiation, aging and the skin: Prevention of damage by topical cAMP manipulation. Molecules 19 (5), 6202–6219. 10.3390/molecules19056202 24838074 PMC4344124

[B2] Anderson A. Bowman A. Boulton S. J. Manning P. Birch-Machin M. A. (2014). A role for human mitochondrial complex II in the production of reactive oxygen species in human skin. Redox Biol. 2, 1016–1022. 10.1016/j.redox.2014.08.005 25460738 PMC4215388

[B3] Avadhani K. S. Manikkath J. Tiwari M. Chandrasekhar M. Godavarthi A. Vidya S. M. (2017). Skin delivery of epigallocatechin-3-gallate (EGCG) and hyaluronic acid loaded nano-transfersomes for antioxidant and anti-aging effects in UV radiation induced skin damage. Drug Deliv. 24 (1), 61–74. 10.1080/10717544.2016.1228718 28155509 PMC8253143

[B4] Barger-Lux M. J. Heaney R. P. (2002). Effects of above average summer sun exposure on serum 25-hydroxyvitamin D and calcium absorption. J. Clin. Endocrinol. Metab. 87 (11), 4952–4956. 10.1210/jc.2002-020636 12414856

[B5] Baroni A. Buommino E. De Gregorio V. Ruocco E. Ruocco V. Wolf R. (2012). Structure and function of the epidermis related to barrier properties. Clin. Dermatol. 30 (3), 257–262. 10.1016/j.clindermatol.2011.08.007 22507037

[B6] Battie C. Jitsukawa S. Bernerd F. Del Bino S. Marionnet C. Verschoore M. (2014). New insights in photoaging, UVA induced damage and skin types. Exp. Dermatol. 23 (1), 7–12. 10.1111/exd.12388 25234829

[B7] Ben-Yehuda Greenwald M. Frušić-Zlotkin M. Soroka Y. Ben-Sasson S. Bianco-Peled H. Kohen R. (2017). A novel role of topical iodine in skin: Activation of the Nrf2 pathway. Free Radic. Biol. Med. 104, 238–248. 10.1016/j.freeradbiomed.2017.01.011 28088623

[B8] Bergeron R. Ren J. M. Cadman K. S. Moore I. K. Perret P. Pypaert M. (2001). Chronic activation of AMP kinase results in NRF-1 activation and mitochondrial biogenesis. Am. J. Physiol. Endocrinol. Metab. 281 (6), E1340–E1346. 10.1152/ajpendo.2001.281.6.E1340 11701451

[B9] Bianconi E. Piovesan A. Facchin F. Beraudi A. Casadei R. Frabetti F. (2013). An estimation of the number of cells in the human body. Ann. Hum. Biol. 40 (6), 463–471. 10.3109/03014460.2013.807878 23829164

[B10] Birch-Machin M. A. Russell E. V. Latimer J. A. (2013). Mitochondrial DNA damage as a biomarker for ultraviolet radiation exposure and oxidative stress. Br. J. Dermatol. 169 (2), 9–14. 10.1111/bjd.12207 23786615

[B11] Birket M. J. Passos J. F. von Zglinicki T. Birch-Machin M. A. (2009). The relationship between the aging- and photo-dependent T414G mitochondrial DNA mutation with cellular senescence and reactive oxygen species production in cultured skin fibroblasts. J. Invest. Dermatol. 129 (6), 1361–1366. 10.1038/jid.2008.373 19052564

[B12] Bosch R. Philips N. Suárez-Pérez J. A. Juarranz A. Devmurari A. Chalensouk-Khaosaat J. (2015). Mechanisms of photoaging and cutaneous photocarcinogenesis, and photoprotective strategies with phytochemicals. Antioxidants (Basel) 4 (2), 248–268. 10.3390/antiox4020248 26783703 PMC4665475

[B13] Cadet J. Grand A. Douki T. (2015). Solar UV radiation-induced DNA bipyrimidine photoproducts: formation and mechanistic insights. Top. Curr. Chem. 356, 249–275. 10.1007/128_2014_553 25370518

[B14] Campisi J. d'Adda di Fagagna F. (2007). Cellular senescence: When bad things happen to good cells. Nat. Rev. Mol. Cell Biol. 8 (9), 729–740. 10.1038/nrm2233 17667954

[B15] Cantó C. Auwerx J. (2009). PGC-1alpha, SIRT1 and AMPK, an energy sensing network that controls energy expenditure. Curr. Opin. Lipidol. 20 (2), 98–105. 10.1097/MOL.0b013e328328d0a4 19276888 PMC3627054

[B16] Cantó C. Jiang L. Q. Deshmukh A. S. Mataki C. Coste A. Lagouge M. (2010). Interdependence of AMPK and SIRT1 for metabolic adaptation to fasting and exercise in skeletal muscle. Cell Metab. 11 (3), 213–219. 10.1016/j.cmet.2010.02.006 20197054 PMC3616265

[B17] Chang C. Su H. Zhang D. Wang Y. Shen Q. Liu B. (2015). AMPK-dependent phosphorylation of GAPDH triggers Sirt1 activation and is necessary for autophagy upon glucose starvation. Mol. Cell 60 (6), 930–940. 10.1016/j.molcel.2015.10.037 26626483

[B18] Chen H. Weng Q. Y. Fisher D. E. (2014). UV signaling pathways within the skin. J. Invest. Dermatol. 134 (8), 2080–2085. 10.1038/jid.2014.161 24759085 PMC4102648

[B19] Cheng T. O. (2006). Danshen: A popular Chinese cardiac herbal drug. J. Am. Coll. Cardiol. 47 (7), 1499–1500. author reply 1499-1500. 10.1016/j.jacc.2006.01.001 16580549

[B20] Chipuk J. E. Moldoveanu T. Llambi F. Parsons M. J. Green D. R. (2010). The BCL-2 family reunion. Mol. Cell 37 (3), 299–310. 10.1016/j.molcel.2010.01.025 20159550 PMC3222298

[B21] Chistiakov D. A. Sobenin I. A. Revin V. V. Orekhov A. N. Bobryshev Y. V. (2014). Mitochondrial aging and age-related dysfunction of mitochondria. Biomed. Res. Int. 2014, 238463. 10.1155/2014/238463 24818134 PMC4003832

[B22] Cueno M. E. Imai K. Tamura M. Ochiai K. (2014). Butyric acid-induced rat jugular blood cytosolic oxidative stress is associated with SIRT1 decrease. Cell Stress Chaperones 19 (2), 295–298. 10.1007/s12192-013-0462-7 24052229 PMC3933618

[B23] de Jager T. L. Cockrell A. E. Du Plessis S. S. (2017). Ultraviolet light induced generation of reactive oxygen species. Adv. Exp. Med. Biol. 996, 15–23. 10.1007/978-3-319-56017-5_2 29124687

[B24] Edlich F. Banerjee S. Suzuki M. Cleland M. M. Arnoult D. Wang C. (2011). Bcl-x(L) retrotranslocates Bax from the mitochondria into the cytosol. Cell 145 (1), 104–116. 10.1016/j.cell.2011.02.034 21458670 PMC3070914

[B25] Fisher G. J. Kang S. Varani J. Bata-Csorgo Z. Wan Y. Datta S. (2002). Mechanisms of photoaging and chronological skin aging. Arch. Dermatol. 138 (11), 1462–1470. 10.1001/archderm.138.11.1462 12437452

[B26] Fitsiou E. Pulido T. Campisi J. Alimirah F. Demaria M. (2021). Cellular senescence and the senescence-associated secretory phenotype as drivers of skin photoaging. J. Invest. Dermatol. 141 (4), 1119–1126. 10.1016/j.jid.2020.09.031 33349436

[B27] Gao S. Guo K. Chen Y. Zhao J. Jing R. Wang L. (2021). Keratinocyte growth factor 2 ameliorates UVB-induced skin damage via activating the AhR/nrf2 signaling pathway. Front. Pharmacol. 12, 655281. 10.3389/fphar.2021.655281 34163354 PMC8215442

[B28] Gęgotek A. Jastrząb A. Jarocka-Karpowicz I. Muszyńska M. Skrzydlewska E. (2018). The effect of sea buckthorn (hippophae rhamnoides L.) seed oil on UV-induced changes in lipid metabolism of human skin cells. Antioxidants (Basel) 7 (9), E110. 10.3390/antiox7090110 30142919 PMC6162715

[B29] Guo X. Kesimer M. Tolun G. Zheng X. Xu Q. Lu J. (2012). The NAD(+)-dependent protein deacetylase activity of SIRT1 is regulated by its oligomeric status. Sci. Rep. 2, 640. 10.1038/srep00640 22962634 PMC3435561

[B30] Guo X. Williams J. G. Schug T. T. Li X. (2010). DYRK1A and DYRK3 promote cell survival through phosphorylation and activation of SIRT1. J. Biol. Chem. 285 (17), 13223–13232. 10.1074/jbc.M110.102574 20167603 PMC2857074

[B31] Hamanaka R. B. Chandel N. S. (2009). Mitochondrial reactive oxygen species regulate hypoxic signaling. Curr. Opin. Cell Biol. 21 (6), 894–899. 10.1016/j.ceb.2009.08.005 19781926 PMC2787901

[B32] Hanasoge S. Ljungman M. (2007). H2AX phosphorylation after UV irradiation is triggered by DNA repair intermediates and is mediated by the ATR kinase. Carcinogenesis 28 (11), 2298–2304. 10.1093/carcin/bgm157 17615256

[B33] Huang X. Traganos F. Darzynkiewicz Z. (2003). DNA damage induced by DNA topoisomerase I- and topoisomerase II-inhibitors detected by histone H2AX phosphorylation in relation to the cell cycle phase and apoptosis. Cell Cycle 2 (6), 613–618. 10.4161/cc.2.6.565 14504478

[B34] Huang Y. H. Chen M. H. Guo Q. L. Chen Z. X. Chen Q. D. Wang X. Z. (2020). Interleukin-10 induces senescence of activated hepatic stellate cells via STAT3-p53 pathway to attenuate liver fibrosis. Cell. Signal. 66, 109445. 10.1016/j.cellsig.2019.109445 31730896

[B35] Hudson L. Bowman A. Rashdan E. Birch-Machin M. A. (2016). Mitochondrial damage and ageing using skin as a model organ. Maturitas 93, 34–40. 10.1016/j.maturitas.2016.04.021 27215947

[B36] Ikehata H. Yamamoto M. (2018). Roles of the KEAP1-NRF2 system in mammalian skin exposed to UV radiation. Toxicol. Appl. Pharmacol. 360, 69–77. 10.1016/j.taap.2018.09.038 30268578

[B37] Kaspar J. W. Niture S. K. Jaiswal A. K. (2009). Nrf2:INrf2 (Keap1) signaling in oxidative stress. Free Radic. Biol. Med. 47 (9), 1304–1309. 10.1016/j.freeradbiomed.2009.07.035 19666107 PMC2763938

[B38] Kawashima S. Funakoshi T. Sato Y. Saito N. Ohsawa H. Kurita K. (2018). Protective effect of pre- and post-vitamin C treatments on UVB-irradiation-induced skin damage. Sci. Rep. 8 (1), 16199. 10.1038/s41598-018-34530-4 30385817 PMC6212420

[B39] Knatko E. V. Ibbotson S. H. Zhang Y. Higgins M. Fahey J. W. Talalay P. (2015). Nrf2 activation protects against solar-simulated ultraviolet radiation in mice and humans. Cancer Prev. Res. 8 (6), 475–486. 10.1158/1940-6207.Capr-14-0362 PMC4454593 25804610

[B40] Krutmann J. Schroeder P. (2009). Role of mitochondria in photoaging of human skin: The defective powerhouse model. J. Investig. Dermatol. Symp. Proc. 14 (1), 44–49. 10.1038/jidsymp.2009.1 19675552

[B41] Lee D. S. Lee S. H. Noh J. G. Hong S. D. (1999). Antibacterial activities of cryptotanshinone and dihydrotanshinone I from a medicinal herb, Salvia miltiorrhiza Bunge. Biosci. Biotechnol. Biochem. 63 (12), 2236–2239. 10.1271/bbb.63.2236 10664860

[B42] Lee J. E. Sim H. Yoo H. M. Lee M. Baek A. Jeon Y. J. (2020). Neuroprotective effects of cryptotanshinone in a direct reprogramming model of Parkinson's disease. Molecules 25 (16), E3602. 10.3390/molecules25163602 32784741 PMC7463464

[B43] Lephart E. D. (2016). Skin aging and oxidative stress: Equol's anti-aging effects via biochemical and molecular mechanisms. Ageing Res. Rev. 31, 36–54. 10.1016/j.arr.2016.08.001 27521253

[B44] Li G. H. Li Y. R. Jiao P. Zhao Y. Hu H. X. Lou H. X. (2018). Therapeutic Potential of Salviae Miltiorrhizae Radix et Rhizoma against Human Diseases Based on Activation of Nrf2-Mediated Antioxidant Defense System: Bioactive Constituents and Mechanism of Action. Oxid. Med. Cell. Longev. 2018, 7309073. 10.1155/2018/7309073 30050659 PMC6040253

[B45] Li H. Jiang N. Liang B. Liu Q. Zhang E. Peng L. (2017). Pterostilbene protects against UVB-induced photo-damage through a phosphatidylinositol-3-kinase-dependent Nrf2/ARE pathway in human keratinocytes. Redox Rep. 22 (6), 501–507. 10.1080/13510002.2017.1329917 28532341 PMC8900625

[B46] Li Y. Shi S. Gao J. Han S. Wu X. Jia Y. (2016). Cryptotanshinone downregulates the profibrotic activities of hypertrophic scar fibroblasts and accelerates wound healing: A potential therapy for the reduction of skin scarring. Biomed. Pharmacother. 80, 80–86. 10.1016/j.biopha.2016.03.006 27133042

[B47] Li Z. Jiang T. Lu Q. Xu K. He J. Xie L. (2020). Berberine attenuated the cytotoxicity induced by t-BHP via inhibiting oxidative stress and mitochondria dysfunction in PC-12 cells. Cell. Mol. Neurobiol. 40 (4), 587–602. 10.1007/s10571-019-00756-7 31828466 PMC11448801

[B48] Liu G. Y. Moon S. H. Jenkins C. M. Li M. Sims H. F. Guan S. (2017). The phospholipase iPLA(2)γ is a major mediator releasing oxidized aliphatic chains from cardiolipin, integrating mitochondrial bioenergetics and signaling. J. Biol. Chem. 292 (25), 10672–10684. 10.1074/jbc.M117.783068 28442572 PMC5481572

[B49] Maione F. Mascolo N. (2016). Danshen and the cardiovascular system: New advances for an old remedy. Semin. Thromb. Hemost. 42 (3), 321–322. 10.1055/s-0036-1580086 26951502

[B50] Makrantonaki E. Zouboulis C. C. (2007). William J. Cunliffe Scientific Awards. Characteristics and pathomechanisms of endogenously aged skin. Dermatology 214 (4), 352–360. 10.1159/000100890 17460411

[B51] Masaki H. Okano Y. Sakurai H. (1999). Generation of active oxygen species from advanced glycation end-products (AGEs) during ultraviolet light A (UVA) irradiation and a possible mechanism for cell damaging. Biochim. Biophys. Acta 1428 (1), 45–56. 10.1016/s0304-4165(99)00056-2 10366759

[B52] Milone M. Massie R. (2010). Polymerase gamma 1 mutations: Clinical correlations. Neurologist 16 (2), 84–91. 10.1097/NRL.0b013e3181c78a89 20220442

[B53] Nagappan A. Kim J. H. Jung D. Y. Jung M. H. (2019). Cryptotanshinone from the Salvia miltiorrhiza Bunge attenuates ethanol-induced liver injury by activation of AMPK/SIRT1 and Nrf2 signaling pathways. Int. J. Mol. Sci. 21 (1), E265. 10.3390/ijms21010265 31906014 PMC6981483

[B54] Oldoni T. L. Melo P. S. Massarioli A. P. Moreno I. A. Bezerra R. M. Rosalen P. L. (2016). Bioassay-guided isolation of proanthocyanidins with antioxidant activity from peanut (*Arachis hypogaea*) skin by combination of chromatography techniques. Food Chem. 192, 306–312. 10.1016/j.foodchem.2015.07.004 26304352

[B55] Orrenius S. Gogvadze V. Zhivotovsky B. (2007). Mitochondrial oxidative stress: Implications for cell death. Annu. Rev. Pharmacol. Toxicol. 47, 143–183. 10.1146/annurev.pharmtox.47.120505.105122 17029566

[B56] Oyewole A. O. Wilmot M. C. Fowler M. Birch-Machin M. A. (2014). Comparing the effects of mitochondrial targeted and localized antioxidants with cellular antioxidants in human skin cells exposed to UVA and hydrogen peroxide. Faseb J. 28 (1), 485–494. 10.1096/fj.13-237008 24115050

[B57] Pang H. Wu L. Tang Y. Zhou G. Qu C. Duan J. A. (2016). Chemical Analysis of the Herbal Medicine Salviae miltiorrhizae Radix et Rhizoma (Danshen). Molecules 21 (1), 51. 10.3390/molecules21010051 26742026 PMC6273254

[B58] Park Y. M. Park S. N. (2019). Inhibitory effect of lupeol on MMPs expression using aged fibroblast through repeated UVA irradiation. Photochem. Photobiol. 95 (2), 587–594. 10.1111/php.13022 30257039

[B59] Passos J. F. Saretzki G. Ahmed S. Nelson G. Richter T. Peters H. (2007). Mitochondrial dysfunction accounts for the stochastic heterogeneity in telomere-dependent senescence. PLoS Biol. 5 (5), e110. 10.1371/journal.pbio.0050110 17472436 PMC1858712

[B60] Picard M. Wallace D. C. Burelle Y. (2016). The rise of mitochondria in medicine. Mitochondrion 30, 105–116. 10.1016/j.mito.2016.07.003 27423788 PMC5023480

[B61] Prinzinger R. (2005). Programmed ageing: The theory of maximal metabolic scope. How does the biological clock tick? EMBO Rep. 6 Spec No (1), S14–S19. 10.1038/sj.embor.7400425 15995655 PMC1369273

[B62] Ravanat J. L. Douki T. Cadet J. (2001). Direct and indirect effects of UV radiation on DNA and its components. J. Photochem. Photobiol. B 63 (1-3), 88–102. 10.1016/s1011-1344(01)00206-8 11684456

[B63] Röck K. Fischer J. W. (2011). Role of the extracellular matrix in extrinsic skin aging. Hautarzt. 62 (8), 591–597. 10.1007/s00105-011-2133-x 21681543

[B64] Rojo de la Vega M. Zhang D. D. Wondrak G. T. (2018). Topical bixin confers NRF2-dependent protection against photodamage and hair graying in mouse skin. Front. Pharmacol. 9, 287. 10.3389/fphar.2018.00287 29636694 PMC5880955

[B65] Sanches Silveira J. E. Myaki Pedroso D. M. (2014). UV light and skin aging. Rev. Environ. Health 29 (3), 243–254. 10.1515/reveh-2014-0058 25241726

[B66] Saw C. L. Huang M. T. Liu Y. Khor T. O. Conney A. H. Kong A. N. (2011). Impact of Nrf2 on UVB-induced skin inflammation/photoprotection and photoprotective effect of sulforaphane. Mol. Carcinog. 50 (6), 479–486. 10.1002/mc.20725 21557329

[B67] Saw C. L. Yang A. Y. Huang M. T. Liu Y. Lee J. H. Khor T. O. (2014). Nrf2 null enhances UVB-induced skin inflammation and extracellular matrix damages. Cell Biosci. 4, 39. 10.1186/2045-3701-4-39 25228981 PMC4164960

[B68] Schon E. A. DiMauro S. Hirano M. (2012). Human mitochondrial DNA: Roles of inherited and somatic mutations. Nat. Rev. Genet. 13 (12), 878–890. 10.1038/nrg3275 23154810 PMC3959762

[B69] Sedlackova L. Korolchuk V. I. (2019). Mitochondrial quality control as a key determinant of cell survival. Biochim. Biophys. Acta. Mol. Cell Res. 1866 (4), 575–587. 10.1016/j.bbamcr.2018.12.012 30594496

[B70] Singh B. Schoeb T. R. Bajpai P. Slominski A. Singh K. K. (2018). Reversing wrinkled skin and hair loss in mice by restoring mitochondrial function. Cell Death Dis. 9 (7), 735. 10.1038/s41419-018-0765-9 30026579 PMC6053453

[B71] Sinha K. Das J. Pal P. B. Sil P. C. (2013). Oxidative stress: The mitochondria-dependent and mitochondria-independent pathways of apoptosis. Arch. Toxicol. 87 (7), 1157–1180. 10.1007/s00204-013-1034-4 23543009

[B72] Stewart J. B. Chinnery P. F. (2015). The dynamics of mitochondrial DNA heteroplasmy: Implications for human health and disease. Nat. Rev. Genet. 16 (9), 530–542. 10.1038/nrg3966 26281784

[B73] Tang L. He S. Wang X. Liu H. Zhu Y. Feng B. (2018). Cryptotanshinone reduces psoriatic epidermal hyperplasia via inhibiting the activation of STAT3. Exp. Dermatol. 27 (3), 268–275. 10.1111/exd.13511 29427477

[B74] Tang Z. Tong X. Huang J. Liu L. Wang D. Yang S. (2021). Research progress of keratinocyte-programmed cell death in UV-induced Skin photodamage. Photodermatol. Photoimmunol. Photomed. 37 (5), 442–448. 10.1111/phpp.12679 33738849

[B75] Uittenbogaard M. Chiaramello A. (2014). Mitochondrial biogenesis: A therapeutic target for neurodevelopmental disorders and neurodegenerative diseases. Curr. Pharm. Des. 20 (35), 5574–5593. 10.2174/1381612820666140305224906 24606804 PMC4823001

[B76] van Erp P. E. Boezeman J. B. Brons P. P. (1996). Cell cycle kinetics in normal human skin by *in vivo* administration of iododeoxyuridine and application of a differentiation marker--implications for cell cycle kinetics in psoriatic skin. Anal. Cell. Pathol. 11 (1), 43–54. 8844104

[B77] Vo T. T. Letai A. (2010). BH3-only proteins and their effects on cancer. Adv. Exp. Med. Biol. 687, 49–63. 10.1007/978-1-4419-6706-0_3 20919637 PMC3733261

[B78] Wang A. S. Dreesen O. (2018). Biomarkers of cellular senescence and skin aging. Front. Genet. 9, 247. 10.3389/fgene.2018.00247 30190724 PMC6115505

[B79] Wang W. Guan C. Sun X. Zhao Z. Li J. Fu X. (2016). Tanshinone IIA protects against acetaminophen-induced hepatotoxicity via activating the Nrf2 pathway. Phytomedicine 23 (6), 589–596. 10.1016/j.phymed.2016.02.022 27161400

[B80] Wang X. Morris-Natschke S. L. Lee K. H. (2007). New developments in the chemistry and biology of the bioactive constituents of Tanshen. Med. Res. Rev. 27 (1), 133–148. 10.1002/med.20077 16888751

[B81] Wang Y. Liang Y. Vanhoutte P. M. (2011). SIRT1 and AMPK in regulating mammalian senescence: A critical review and a working model. FEBS Lett. 585 (7), 986–994. 10.1016/j.febslet.2010.11.047 21130086

[B82] Wiley C. D. Velarde M. C. Lecot P. Liu S. Sarnoski E. A. Freund A. (2016). Mitochondrial dysfunction induces senescence with a distinct secretory phenotype. Cell Metab. 23 (2), 303–314. 10.1016/j.cmet.2015.11.011 26686024 PMC4749409

[B83] Wong R. Geyer S. Weninger W. Guimberteau J. C. Wong J. K. (2016). The dynamic anatomy and patterning of skin. Exp. Dermatol. 25 (2), 92–98. 10.1111/exd.12832 26284579

[B84] Wu Y. H. Wu Y. R. Li B. Yan Z. Y. (2020). Cryptotanshinone: A review of its pharmacology activities and molecular mechanisms. Fitoterapia 145, 104633. 10.1016/j.fitote.2020.104633 32445662

[B85] Wu Z. Song L. Liu S. Q. Huang D. (2014). Tanshinones extend chronological lifespan in budding yeast *Saccharomyces cerevisiae* . Appl. Microbiol. Biotechnol. 98 (20), 8617–8628. 10.1007/s00253-014-5890-5 24970458

[B86] Ye T. Zhu S. Zhu Y. Feng Q. He B. Xiong Y. (2016). Cryptotanshinone induces melanoma cancer cells apoptosis via ROS-mitochondrial apoptotic pathway and impairs cell migration and invasion. Biomed. Pharmacother. 82, 319–326. 10.1016/j.biopha.2016.05.015 27470369

[B87] Zarei F. Abbaszadeh A. (2019). Application of cell therapy for anti-aging facial skin. Curr. Stem Cell Res. Ther. 14 (3), 244–248. 10.2174/1574888x13666181113113415 30421684

[B88] Zhang R. Wang Y. Ye K. Picard M. Gu Z. (2017). Independent impacts of aging on mitochondrial DNA quantity and quality in humans. BMC Genomics 18 (1), 890. 10.1186/s12864-017-4287-0 29157198 PMC5697406

[B89] Zhang Y. Chen L. Li F. Wang H. Yao Y. Shu J. (2016). Cryptotanshinone protects against adriamycin-induced mitochondrial dysfunction in cardiomyocytes. Pharm. Biol. 54 (2), 237–242. 10.3109/13880209.2015.1029052 25858002

[B90] Zhao G. R. Zhang H. M. Ye T. X. Xiang Z. J. Yuan Y. J. Guo Z. X. (2008). Characterization of the radical scavenging and antioxidant activities of danshensu and salvianolic acid B. Food Chem. Toxicol. 46 (1), 73–81. 10.1016/j.fct.2007.06.034 17719161

[B91] Zhao X. Toyooka T. Ibuki Y. (2014). Silver ions enhance UVB-induced phosphorylation of histone H2AX. Environ. Mol. Mutagen. 55 (7), 556–565. 10.1002/em.21875 24838775

[B92] Zheng X. Chen L. Jin S. Xiong L. Chen H. Hu K. (2021). Ultraviolet B irradiation up-regulates MM1 and induces photoageing of the epidermis. Photodermatol. Photoimmunol. Photomed. 37 (5), 395–403. 10.1111/phpp.12670 33565151

[B93] Zhou L. Zuo Z. Chow M. S. (2005). Danshen: An overview of its chemistry, pharmacology, pharmacokinetics, and clinical use. J. Clin. Pharmacol. 45 (12), 1345–1359. 10.1177/0091270005282630 16291709

[B94] Zhou Y. Wang X. Ying W. Wu D. Zhong P. (2019). Cryptotanshinone attenuates inflammatory response of microglial cells via the Nrf2/HO-1 pathway. Front. Neurosci. 13, 852. 10.3389/fnins.2019.00852 31496930 PMC6712928

[B95] Zhuang X. X. Wang S. F. Tan Y. Song J. X. Zhu Z. Wang Z. Y. (2020). Pharmacological enhancement of TFEB-mediated autophagy alleviated neuronal death in oxidative stress-induced Parkinson's disease models. Cell Death Dis. 11 (2), 128. 10.1038/s41419-020-2322-6 32071296 PMC7028954

[B96] Zong H. Ren J. M. Young L. H. Pypaert M. Mu J. Birnbaum M. J. (2002). AMP kinase is required for mitochondrial biogenesis in skeletal muscle in response to chronic energy deprivation. Proc. Natl. Acad. Sci. U. S. A. 99 (25), 15983–15987. 10.1073/pnas.252625599 12444247 PMC138551

